# Myocardial native T_1_ mapping and extracellular volume quantification in asymptomatic female carriers of Duchenne muscular dystrophy gene mutations

**DOI:** 10.1186/s13023-023-02899-9

**Published:** 2023-09-11

**Authors:** Lucia Masárová, Roman Panovský, Martin Pešl, Mary Luz Mojica-Pisciotti, Tomáš Holeček, Vladimír Kincl, Lenka Juříková, Jan Máchal, Lukáš Opatřil, Věra Feitová

**Affiliations:** 1 International Clinical Research Centre, St. Anne’s University Hospital, Brno, Czech Republic; 2 1st Department of Internal Medicine-Cardioangiology, St. Anne’s University Hospital, Faculty of Medicine, Masaryk University, Brno, Czech Republic; 3 Department of Biology, Faculty of Medicine, Masaryk University, Brno, Czech Republic; 4 Department of Pathophysiology, Faculty of Medicine, Masaryk University, Brno, Czech Republic; 5 Department of Medical Imaging, St. Anne’s University Hospital, Brno, Czech Republic; 6 Department of Paediatric Neurology, University Hospital, Brno, Czech Republic; 7 Department of Biomedical Engineering, University of Technology, Brno, Czech Republic

**Keywords:** Cardiac magnetic resonance, Duchenne muscular dystrophy, Native T_1_ mapping, Extracellular volume quantification, Late gadolinium enhancement

## Abstract

**Background:**

Female carriers of dystrophin gene mutations (DMD-FC) were previously considered non-manifesting, but in recent decades, cardiomyopathy associated with muscular dystrophy and myocardial fibrosis has been described. Our study aimed to assess prospectively myocardial fibrosis in asymptomatic DMD-FC compared to a sex-matched control group (CG) with similar age distribution using native T_1_ mapping and extracellular volume (ECV) quantification by cardiovascular magnetic resonance (CMR) imaging.

**Materials and methods:**

38 DMD-FC with verified genetic mutation and 22 healthy volunteers were included. Using CMR, native T_1_ relaxation time and ECV quantification were determined in each group. Late gadolinium enhancement (LGE) was assessed in all cases.

**Results:**

There were 38 DMD-FC (mean age 39.1 ± 8.8 years) and 22 healthy volunteers (mean age 39.9 ± 12.6 years) imagined by CMR. The mean global native T_1_ relaxation time was similar for DMD-FC and CG (1005.1 ± 26.3 ms vs. 1003.5 ± 25.0 ms; p-value = 0.81). Likewise, the mean global ECV value was also similar between the groups (27.92 ± 2.02% vs. 27.10 ± 2.89%; p-value = 0.20). The segmental analysis of mean ECV values according to the American Heart Association classification did not show any differences between DMD-FC and CG. There was a non-significant trend towards higher mean ECV values of DMD-FC in the inferior and inferolateral segments of the myocardium (p-value = 0.075 and 0.070 respectively).

**Conclusion:**

There were no statistically significant differences in the mean global and segmental native T_1_ relaxation times and the mean global or segmental ECV values. There was a trend towards higher segmental mean ECV values of DMD-FC in the inferior and inferolateral walls of the myocardium.

**Supplementary Information:**

The online version contains supplementary material available at 10.1186/s13023-023-02899-9.

## Introduction

Duchenne (DMD) and Becker (BMD) muscular dystrophies are X-linked recessive disorders caused by mutations in the dystrophin gene. Progressive muscular wasting, weakness, respiratory failure, and cardiovascular diseases are caused by pathogenic variants in DMD patients [1, 2]. It has been found that cardiac complications play a relevant role in muscular dystrophies [3].

The most common form of cardiac involvement in muscular dystrophy is dilated cardiomyopathy [4, 5], presenting as an age-related progression of left ventricular (LV) dysfunction and myocardial fibrosis, which can be detected by late gadolinium enhancement (LGE) cardiovascular magnetic resonance (CMR) imaging [6].

Female carriers of Duchenne muscular dystrophy gene mutations (DMD- healthy patients) were previously considered non-manifesting [5]. However, in recent decades, it has become evident that DMD-FC can be affected similarly, albeit more mildly, than DMD patients (affected males) [7–10]. DMD-FC can present cardiac involvement, such as cardiomyopathy, associated with muscular dystrophy [11, 12]. Although DMD-FC are usually asymptomatic, they also can be affected, similarly to DMD patients, by myocardial fibrosis. A well-established technique for assessing myocardial fibrosis is late gadolinium enhancement (LGE). However, it is limited for identifying interstitial diffuse fibrotic changes in the myocardium [13]. Since it may precede the development of LV dysfunction [11], its assessment is crucial in DMD-FC.

Therefore, other CMR-based methods, such as native T_1_ mapping and extracellular volume (ECV) quantification, might be more suitable for detecting diffuse myocardial fibrosis, as already-published studies in DMD/BMD patients have shown [14–18]. Also, there have been a few published reports about mildly elevated native T_1_ relaxation time or elevated ECV values in DMD/BMD-FC [11, 19, 20].

This study aims to assess prospectively myocardial fibrosis in asymptomatic DMD-FC compared to a sex-matched control group (CG) with similar age distribution using native T_1_ mapping and ECV quantification by CMR. Prior to now, there had been no direct comparison to CG.

## Materials and methods

The demographically similar study population included 38 DMD-FC with verified genetic mutation and 22 healthy volunteers. The most common mutation was the deletion of 45–52 exons. The asymptomatic DMD-FC were defined based on clinical examination, and all completed the prepared questionnaire.

All eligible subjects who fulfilled the inclusion criteria (age over 18 years, signed informed consent, absence of CMR contraindications, and cardiovascular pathology besides dystrophic cardiomyopathies) were enrolled. The volunteers had no pathological findings on CMR, no anamnesis of cardiac disease, and no other pathological tests. Exclusion criteria for both groups included renal insufficiency (estimated glomerular filtration rate < 30 mL/min/1.73 m^2^), CMR contraindications, or limited life expectancy.

The hematocrit was obtained on the same day that CMR was performed. Following the Declaration of Helsinki (2000) of the World Medical Association, the Faculty Hospital St. Anne’s Ethics Committee (reference number 55 V/2016) approved the study.

### CMR acquisition

CMR was performed using a 1.5T scanner (Ingenia, Philips Medical Systems, Best, The Netherlands) according to our standard protocol. It was equipped with 5-element posterior and 32-element anterior phased-array receiver coils allowing for parallel acquisition techniques in the supine position with repeated breath-hold. Functional imaging using balanced turbo field echo (b-TFE) cine sequences included four-chamber, two-chamber, and LV outflow tract long-axis views and a short-axis (SAX) stack. Typical acquisition parameters were: field of view 320 × 280 mm, reconstruction matrix 256, slice thickness 8 mm, acquisition voxel size 1.7 × 1.7 × 8.0 mm, repetition time ≈ 3.2 ms, echo time ≈ 1.6 ms, and SENSE factor 1.7.

LGE images in all long-axis views and the SAX view were acquired 10 min after an intravenous bolus of 0.2 mmol/kg of the gadolinium-based contrast agent gadobutrol (Gadovist, Bayer-Schering Pharma, Germany) using an inversion recovery (IR-TFE) sequence, and, if uncertain, by phase-sensitive inversion recovery TFE (PSIR-TFE). Both two- and three-dimensional acquisitions were performed in mid-diastole. Typical parameters for the IR-TFE sequence were field of view 320 × 320 mm, reconstruction matrix 288, voxel size 1.6 × 1.7 × 10 mm, repetition time ≈ 4.1 ms, echo time ≈ 1.2 ms, and SENSE factor 2.5.

T_1_ mapping was performed as described previously [17] using a Modified Look-Locker Inversion recovery sequence (MOLLI) with a 5(3)3 scheme to measure native T_1_ (pre-contrast) and a 4(1)3(1)2 scheme for T_1_ post-gadolinium (15 min after contrast agent administration). MOLLI sequences were acquired at the mid-ventricular level in the SAX plane using typical imaging parameters (field of view 300 × 300 mm, reconstruction matrix 256, slice thickness 10 mm, acquisition voxel size 2.00 × 2.00 × 10.00 mm, time to repetition ≈ 2.2 ms, echo time ≈ 1.1 ms, flip angle 35°, SENSE factor 2).

### MR data analysis

Two experienced readers (MLMP, TH) used cvi42 (release 5.13.9, Circle Cardiovascular Imaging, Calgary, Canada) to construct the native T_1_ and post-gadolinium T_1_ maps. They manually contoured the epi- and endocardial walls in the mid-ventricular slice of SAX using 10% of the myocardial wall as border cutting in both the native and the post-gadolinium images. Then, a motion correction algorithm (integrated into cvi42) was applied to register the images, and the software calculated pixel-by-pixel maps from these images. The ECV quantification was calculated according to (1-hematocrit) (1/T1myo,post – 1/T1myo,native)/(1/T1blood,post – 1/T1blood,native) for each segment, and the mean global ECV value was the average of the values in those segments [21]. The acronyms of the mentioned formula represent: the native and post-gadolinium T_1_ values of the myocardium/blood before and after the application of the contrast agent.

Wall motion abnormalities were assessed qualitatively (visually) by an experienced cardiologist (RP) with 29 years of experience and a radiologist (VF) with 38 years of experience with CMR. LV functional and morphological parameters were calculated from the SAX stack using the summation-of-disc method following the recommendations on post-processing evaluation from the Society for Cardiovascular Magnetic Resonance (SCMR) [22].

The radiologist (VF) and the cardiologist (RP) employed a semi-quantitative approach to determine the presence of LGE according to the American Heart Association (AHA) 17-segment model [23] in the IntelliSpace Portal workspace (version 11, Philips Healthcare). LGE was defined as positive if the visual enhancement was higher than the mean signal intensity of the reference myocardium, a remote or unaffected myocardial region within the same patient. A DMD-FC with LGE in at least one myocardial segment was considered LGE-positive. If no enhancement was observed, the DMD-FC was identified as LGE-negative.

### Statistics

Variables in both groups were compared using the Student’s t-test for unpaired data. The normal distribution was checked by the Kolmogorov-Smirnov test and a visual inspection of histograms; in a case when the Student’s t-test revealed no difference, this meant the Kolmogorov-Smirnov test was also used to check the identity of distributions of quantitative variables in both groups. The data are presented as mean ± standard deviation (SD).

The power of the tests was considered for native T_1_ relaxation time and ECV values to determine the magnitude of the effect that could be identified as statistically significant by the Student’s t-test. The following assumptions were used: α = 0.05; power = 0.8 (0.6, respectively); normal distribution; standard deviation corresponding with the actual distribution in the CG. As the nature of our study was explorative, we neither assumed nor performed any multiple testing corrections, which would increase the risk of type II error and decrease the power of the test.

The interobserver agreement of both native and post-gadolinium global T_1_ relaxation time was assessed with the intraclass correlation coefficient (ICC, two-way mixed-effects model), which was determined from eight randomly selected cases analysed by two readers (MLMP, TH).

All analyses were performed using Statistica (version 14.0. TIBCO Software Inc., 2020) and in R (v4.2.1) with RStudio IDE (v2022.7.1.554, RStudio, PBC) software. The value of α = 0.05 was used as a threshold for statistical significance throughout the analyses.

## Results

38 DMD-FC (mean age 39.1 ± 8.8 years) and 22 healthy volunteers (mean age 39.9 ± 12.6 years) were included. There was no statistically significant difference between the LVEF of DMD-FC (65.7 ± 5.5%) and CG (68.4 ± 6.57%; p = 0.09). 5% of asymptomatic DMD-FC of our cohort had small hypokinesia of the apex and the inferolateral wall of LV. Also, 20% of DMD-FC had LGE that was detected on the inferolateral wall (4 DMD-FC) and inferior wall (3 DMD-FC) of LV, while LGE was absent in the CG.

**Fig. 1:**
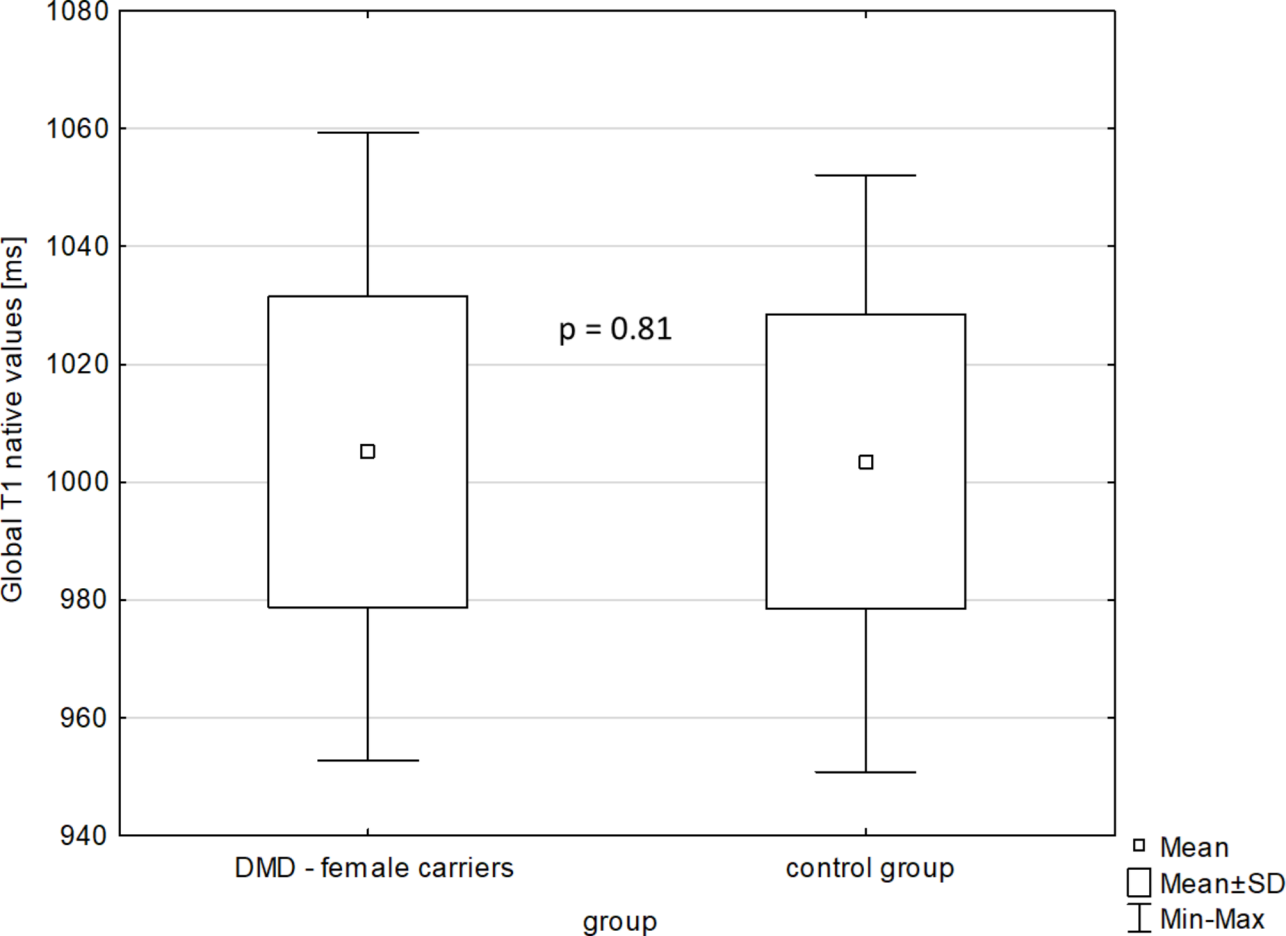
The mean global native T_**1**_ relaxation time values [ms] in DMD-FC and CG. DMD- FC = Female carriers of Duchenne muscular dystrophy gene mutations; CG = Control group

**Fig. 2:**
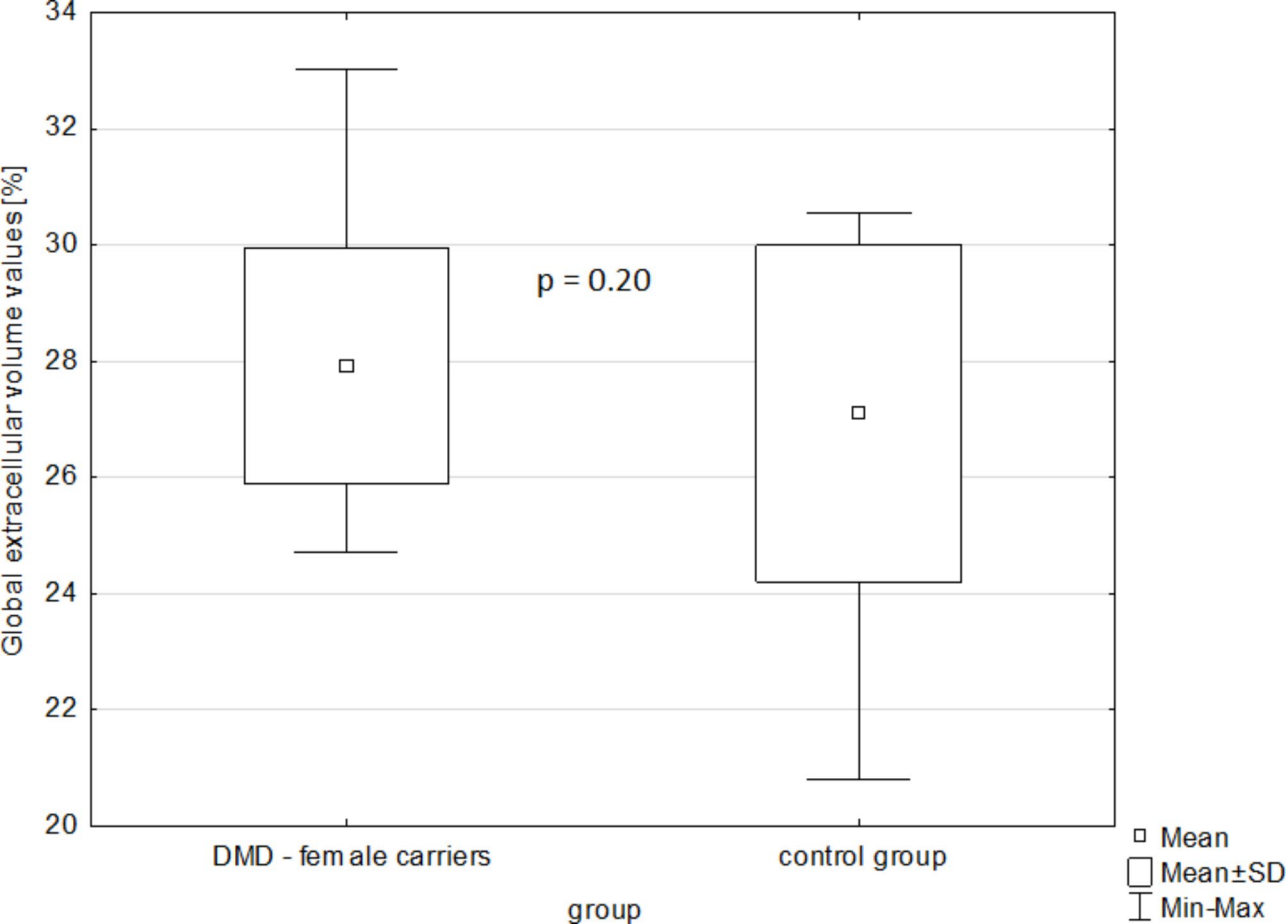
The mean global extracellular volume values [%] in DMD-FC and CG. DMD- FC = Female carriers of Duchenne muscular dystrophy gene mutations; CG = Control group

**Fig. 3:**
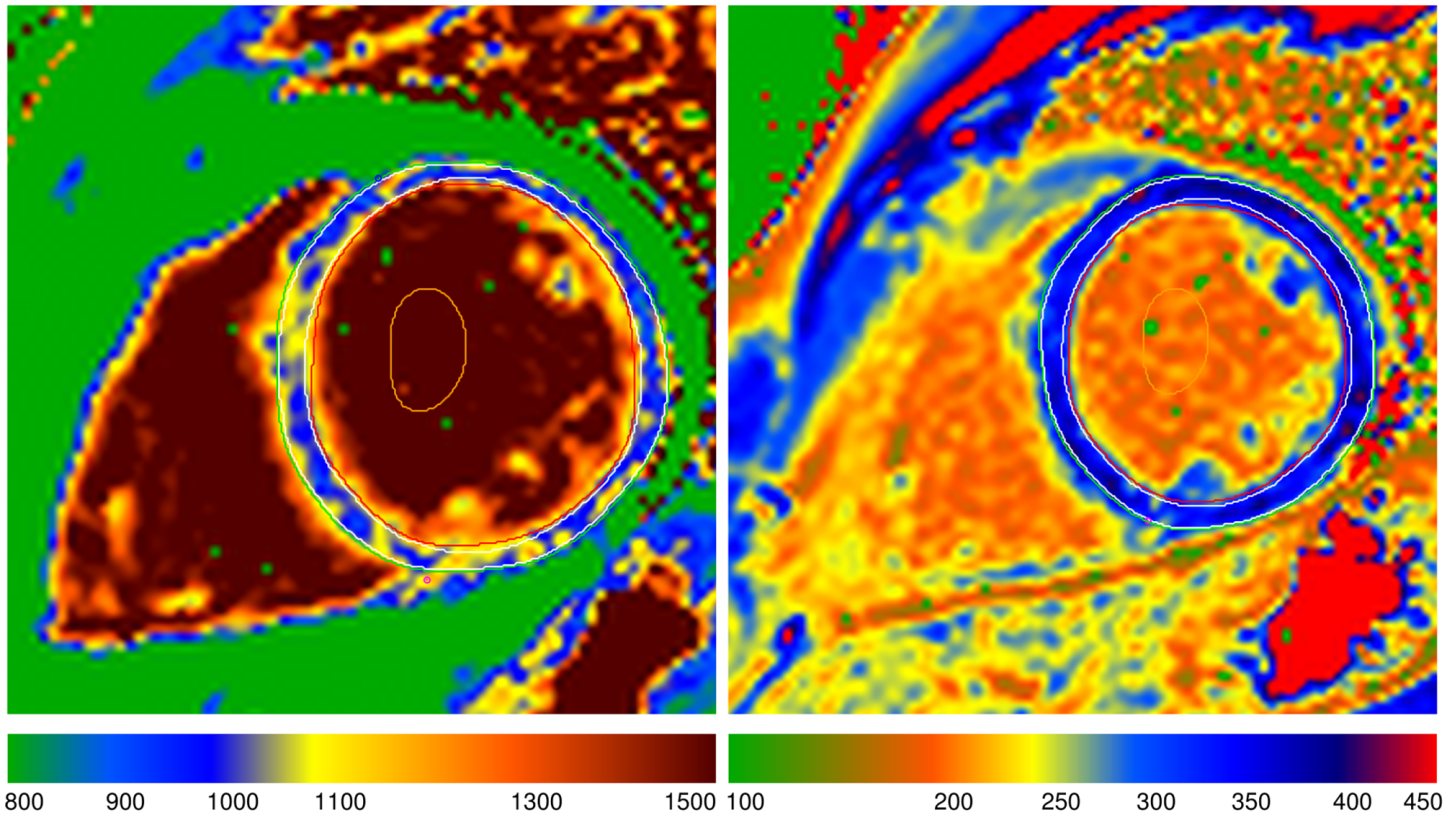
A representative picture of the native and post-gadolinium T_1_ map of DMD-FC. DMD-FC - Female carriers of Duchenne muscular dystrophy gene mutations

**Fig. 4:**
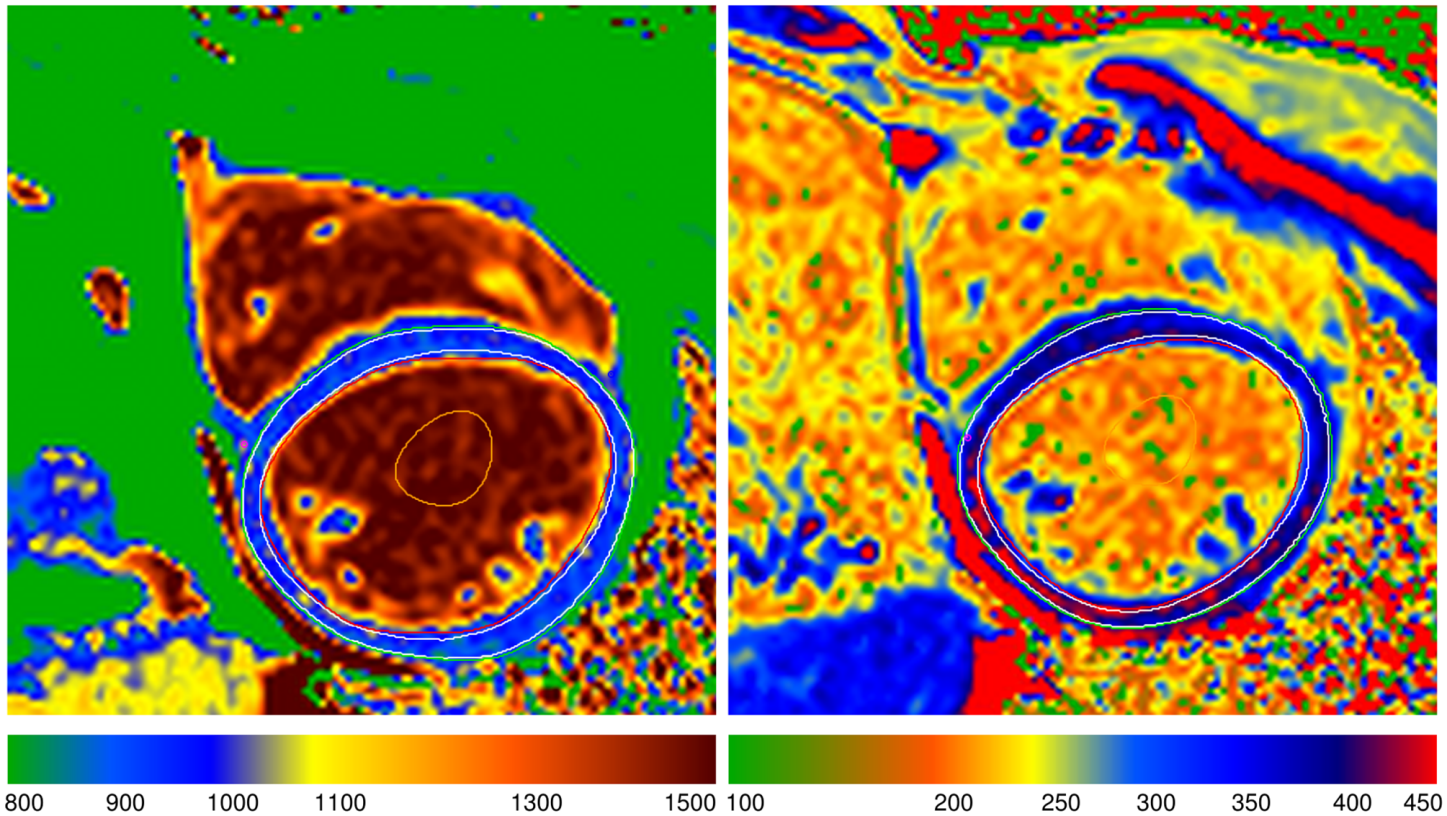
A representative picture of the native and post-gadolinium T_1_ map of a healthy volunteer

The mean global native T_1_ relaxation time was similar for DMD-FC and CG (1005.1 ± 26.3 ms vs. 1003.5 ± 25 ms; p-value = 0.81) (Fig. 1), as well as the mean global ECV value (27.92 ± 2.02% vs. 27.09 ± 2.89%; p-value = 0.20) (Fig. 2). The representative native and post-gadolinium T_1_ maps are presented in Fig. 3 for DMD-FC and Fig. 4 for healthy volunteer. No statistically significant differences were detected between DMD-FC with LGE positive and LGE negative in the mean ECV segmental values. The ECV segmental quantification analysis, according to the AHA classification, did not show any differences for DMD-FC vs. CG. Although statistically non-significant, there was a trend towards higher mean ECV values in the inferior (p = 0.075) and inferolateral (p = 0.070) segments of the myocardium in DMD-FC. The detailed results are shown in Table 1. When the identical distribution of measured variables in DMD-FC and in CG was checked by Kolmogorov-Smirnov test, it yielded a p-value > 0.10 in all cases, including mean global and segmental T_1_ relaxation times and ECV values, age and LVEF.

**Table 1:** Comparison of mean global and segmental native T_1_ relaxation time and mean global and segmental ECV values and other parameters between DMD-FC and the CG

Parameter	DMD-FC (n = 38)	CG (n = 22)	P-value
**Age [years]**	39 ± 8.8	39.9 ± 12.6	0.76
**BMI [kg/m** ^**2**^ **]**	23.8 ± 3.4	23.17 ± 3.9	0.64
**LVEF [%]**	65.6 ± 5.5	68.4 ± 6.57	0.33
**Global native T**_**1 **_ **relaxation time [ms]**	1005.1 ± 26.3	1003.5 ± 25.0	0.81
**Native T**_**1 **_ **relaxation time [ms]: anterior segment**	997.3 ± 33.7	1006.7 ± 35.9	0.31
**Native T**_**1 **_ **relaxation time [ms]: anteroseptal segment**	1001.5 ± 35.2	994.9 ± 26.9	0.45
**Native T**_**1 **_ **relaxation time [ms]: inferoseptal segment**	1013.8 ± 26.0	1004.5 ± 30.8	0.22
**Native T**_**1 **_ **relaxation time [ms]: inferior segment**	1008.7 ± 40.1	1003.5 ± 31.9	0.60
**Native T**_**1 **_ **relaxation time [ms]: inferolateral segment**	1012.5 ± 32.4	1012.4 ± 36.7	0.99
**Native T**_**1 **_ **relaxation time [ms]: anterolateral segment**	992.8 ± 32.0	1004.7 ± 37.0	0.19
**Global ECV value [%]**	27.93 ± 2.02	27.10 ± 2.89	0.20
**ECV value: anterior segment**	27.86 ± 2.35	26.90 ± 2.66	0.16
**ECV value: anteroseptal segment**	27.72 ± 2.10	27.62 ± 3.17	0.88
**ECV value: inferoseptal segment**	28.41 ± 2.21	27.73 ± 3.25	0.34
**ECV value: inferior segment**	28.26 ± 2.62	26.79 ± 3.65	0.075
**ECV value: inferolateral segment**	27.83 ± 2.94	26.30 ± 3.34	0.070
**ECV value: anterolateral segment**	27.63 ± 2.30	27.02 ± 3.12	0.39

Parameters are shown as mean ± standard deviation

BMI = body mass index; ECV = extracellular volume; DMD- FC = Female carriers of Duchenne muscular dystrophy gene mutations; CG = Control group; LVEF = left ventricle ejection fraction

Regarding the global native T_1_ relaxation time, the sample size was sufficiently large to statistically prove a difference of 19 ms with a power of 80% and a difference of 15 ms with a power of 60%. In the case of ECV values, the sample size enabled the discrimination of values differing by 2.3% with a power of 80% and a difference of 1.8% with a power of 60%.

The values of ICC were 0.980 (95% CI: 0.921 to 0.995) for mean global native T_1_ and 0.976 (95% CI: 0.906 to 0.994) for mean global T_1_ post-gadolinium relaxation times, showing excellent interobserver reliability.

## Discussion

There were no statistically significant differences in the mean global and segmental native T_1_ relaxation times or the mean global and segmental ECV values in asymptomatic DMD-FC with normal LVEF compared to sex -matched CG with similar age distribution. Regarding the segmental values, although statistically non-significant, there was a trend towards higher mean ECV values in the inferior and inferolateral segments of the myocardium in DMD-FC in our study. This may be potentially of concern, as similar segments are affected as in DMD patients; however it should be noted that the magnitude of those differences is much smaller than of those observed between DMD patients and CG, as is evident in various cited studies [15, 16] including ours [17], which correspond to the typically affected regions in the muscular dystrophies.

Cardiac involvement, such as cardiomyopathy and the presence of LGE in typical localization, was recently described in DMD-FC [11, 12]. To date, there have been only 3 published works focused on native T_1_ mapping and ECV quantification in DMD/BMD-FC [11, 19, 20], of which only one used a case-control design.

The first of the studies describes diffuse myocardial fibrosis assessed by ECV quantification in DMD/BMD-FC of gene mutations for muscular dystrophies [20]. Analysed were 5 DMD/BMD-FC ranging from 43.0 to 51.7 years. It showed elevated global ECV values of 37.4% in DMD/BMD-FC and BMD/DMD/myotonic dystrophic patients regardless of the presence of LGE. All 5 screened DMD/BMD-FC were older than DMD-FC in our study. Moreover, one of them had reduced LVEF, 2 of them were LGE positive, and there was also a combination of DMD and BMD-FC.

The second one [11] also reported mildly abnormal ECV values in DMD-FC.

The last study compared the DMD-FC to non-carrier female relatives and CG and found higher native T_1_ relaxation time in DMD/BMD-FC compared to non-carriers and CG [19]; however this study did not assess the ECV quantification or regional differences in native T_1_ relaxation time. While our study had sufficient power to reveal the differences in global native T_1_ relaxation time with a size comparable to [19] and employed study subjects of a comparable age, it did not confirm the increase in native T_1_ relaxation time found in this study.

The main contribution of our study compared to the mentioned studies [11, 12, 19, 20] is the evaluation of the diffuse fibrotic changes of the myocardium using native T_1_ mapping and ECV quantification in a much larger cohort of DMD-FC, and with regards to CG. Moreover, we focused on the assessment of more than just the presence of LGE in the typical location or the described mildly elevated value of ECV or global native T_1_ relaxation time. This is the first study that compares global and segmental native T_1_ and ECV values between DMD-FC and CG in a case/control design.

LGE can aid in detecting the extent and the location of myocardial fibrosis. Additionally LGE can uncover incipient heart disease by its presence [14, 24]. Whereas native T_1_ mapping and ECV quantification allow for the measurement of diffuse fibrosis that foregoes the mentioned LGE. These methods are also useful in detecting myocardial inflammation or any alteration of the extracellular space [14, 25].

Contrary to DMD-FC, many more studies have assessed native T_1_ mapping and quantified ECV in DMD patients [16, 26, 27]. Although LGE positive DMD patients exhibited an increased global native T_1_ relaxation time compared to CG, there was no difference between LGE negative DMD patients and CG [27]. Moreover, DMD patients had a significantly increased lateral myocardial native T_1_ relaxation time compared to CG [28].

These results align with other authors who detected increased native T_1_ relaxation time and ECV values in DMD patients. ECV quantification was regardless the ability to differentiate LGE negative DMD patients and CG [29].

Both native T_1_ mapping and ECV quantification can identify the differences and regional abnormalities on the inferolateral wall of the LV between DMD patients and CG with or without LGE [21, 30–33]. Additionally, increased native T_1_ relaxation time and reduced LVEF was reported in LGE positive DMD patients, evidencing a relationship between native T_1_ relaxation time and LV function [28].

Based on our previous studies, all global LV strains and tissue Doppler parameters in asymptomatic DMD-FC were decreased compared to CG [34, 35]. Despite a preserved LVEF, subclinical changes in the LV systolic function were discovered in DMD-FC, including decreased global LV strains. However, their mean global and segmental native T_1_ relaxation times and mean global and segmental ECV values were similar to sex- matched CG with similar age distribution. Furthermore, 20% of our DMD-FC cohort had LGE on the inferolateral wall of LV, which is typical localization for the extracellular volume expansion described in DMD patients [36, 37].

While the differences in native T_1_ relaxation time and ECV quantification in symptomatic DMD patients are already known, our DMD-FC cohort did not have any cardiac symptoms.

### Limitations

This work has some limitations. Some of them are related to the general limitations of native T_1_ mapping [4, 38]. It is also limited by its sample size due to the rare occurrence of DMD disease. It is a single centre study. All the subjects involved in this study, were analysed under the same conditions: the images were acquired with the same equipment and protocol, and the analyses were assessed similarly. The healthy volunteers were not tightly selected, as they were considered healthy because of their lack of CMR-based evidence of cardiac problems. Any possible bias affected all the subjects equally. Although the interobserver agreement was excellent, it was determined only for the mean global native T_1_ relaxation time and mean global ECV values. Future research is essential to validate the whole process’s reproducibility.

## Conclusion

The mean global and segmental native T_1_ relaxation times and mean global and segmental ECV values in asymptomatic DMD-FC with normal LVEF were similar to CG, as there was no statistically significant difference in any of the CMR-assessed parameters. Although non-significant, there was a trend towards higher mean ECV values of DMD-FC in inferior and inferolateral segments of the myocardium, which should be subjected to prospective assessment.

### Electronic supplementary material

Below is the link to the electronic supplementary material.

Supplementary Material 1

## Data Availability

The datasets analysed during the current study are available from the corresponding author upon reasonable request. Data are located in controlled access data storage REDCap at St. Anne’s University Hospital (https://redcap.fnusa.cz/redcap/).

## Abbreviations

AHA: American heart association
BMD: Becker muscular dystrophy
b-TFE: balanced - turbo field echo
CI: Confidence interval
CG: Control group
CMR: Cardiac magnetic resonance
DMD: Duchenne muscular dystrophy
DMD-FC: Female carriers of Duchenne muscular dystrophy gene mutations
ECV: Extracellular volume
EDV: End-diastolic volume
ESV: End-systolic volume
ICC: Intraclass correlation
IR-TFE: Inversion-recovery turbo field echo
LGE: Late gadolinium enhancement
LV: Left ventricle
LVEF: Ejection fraction of left ventricle
PSIR: Phase-sensitive inversion recovery
SAX: Short axis
SCMR: Society for Cardiovascular Magnetic Resonance
SD: Standard deviation
T1myo,post: post-gadolinium T_1_ values of myocardium
T1myo,native: native T_1_ values of myocardium
T1blood,post: post-gadolinium T_1_ values of blood
T1blood,native: native T_1_ values of blood
