# Correction: Myocardial native T_1_ mapping and extracellular volume quantification in asymptomatic female carriers of Duchenne muscular dystrophy gene mutations

**DOI:** 10.1186/s13023-023-02922-z

**Published:** 2023-10-19

**Authors:** Lucia Masárová, Roman Panovský, Martin Pešl, Luz Mojica-Pisciotti Mary, Tomáš Holeček, Vladimír Kincl, Lenka Juříková, Jan Máchal, Lukáš Opatřil, Věra Feitová

**Affiliations:** 1 International Clinical Research Centre, St. Anne’s University Hospital, Brno, Czech Republic; 2 1st Department of Internal Medicine-Cardioangiology, St. Anne’s University Hospital, Faculty of Medicine, Masaryk University, Brno, Czech Republic; 3 Department of Biology, Faculty of Medicine, Masaryk University, Brno, Czech Republic; 4 Department of Pathophysiology, Faculty of Medicine, Masaryk University, Brno, Czech Republic; 5 Department of Medical Imaging, St. Anne’s University Hospital, Brno, Czech Republic; 6 Department of Paediatric Neurology, University Hospital, Brno, Czech Republic; 7 Department of Biomedical Engineering, University of Technology, Brno, Czech Republic

**Correction: Journal of Rare Diseases (2023) 18:283** 10.1186/s13023-023-02899-9

Following publication of the original article [1], we have been notified of several mistakes.

The authors’ names were initially published as per below:

Masárová Lucia1,2, Panovský Roman1,2*, Pešl Martin1,2,3, Mojica-Pisciotti Mary Luz1,2, Holeček Tomáš1,5,7, Kincl Vladimír1,2, Juříková Lenka6, Máchal Jan1,4, Opatřil Lukáš1,2 and Feitová Věra1,5

**They should be as follows:**

Lucia Masárová1,2, Roman Panovský1,2*,Martin Pešl1,2,3, Mary Luz Mojica-Pisciotti1,2, Tomáš Holeček1,5,7, Vladimír Kincl1,2, Lenka Juříková6, Jan Máchal1,4, Lukáš Opatřil1,2 and Věra Feitová1,5

Results section text was as follows (bold and incorrect spacing):

The mean global native T1 relaxation time was similar for DMD-FC and CG (1005.1 ± 26.3 ms vs. 1003.5 ± 25 ms; p-value = 0.81) (Fig. 1), as well as the mean global ECV value (27.92 ± 2.02% vs. 27.09 ± 2.89%; p-value = 0.20) (Fig. 2). The representative native and post-gadolinium T1 maps are presented in Fig. 3f or **DMD-FC** and Fig. 4f or **healthy volunteer.**

## The text should be corrected as follows:

The mean global native T1 relaxation time was similar for DMD-FC and CG (1005.1 ± 26.3 ms vs. 1003.5 ± 25 ms; p-value = 0.81) (Fig. 1), as well as the mean global ECV value (27.92 ± 2.02% vs. 27.09 ± 2.89%; p-value = 0.20) (Fig. 2). The representative native and post-gadolinium T1 maps are presented in Fig. 3 for DMD-FC and Fig. 4 for healthy volunteer.

Figure 3 was incorrect and with incorrect caption as per below:
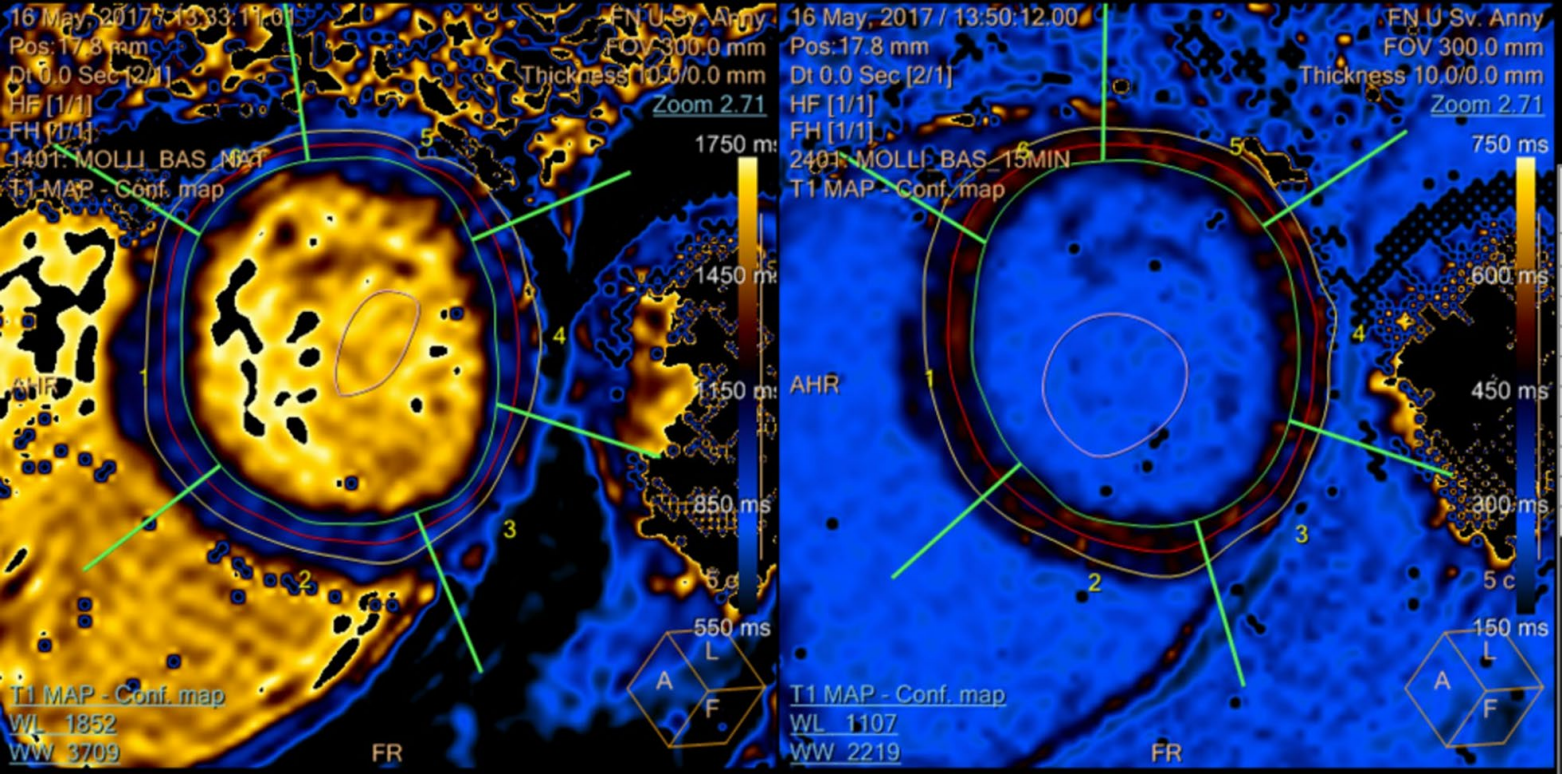


**Fig. 3** A representative picture of the native and post-gadolinium T_1_ map of DMD-FC. DMD-FC- Female carriers of Duchenne muscular dystrophy gene mutations

Figure 3 and its caption should be as follows:
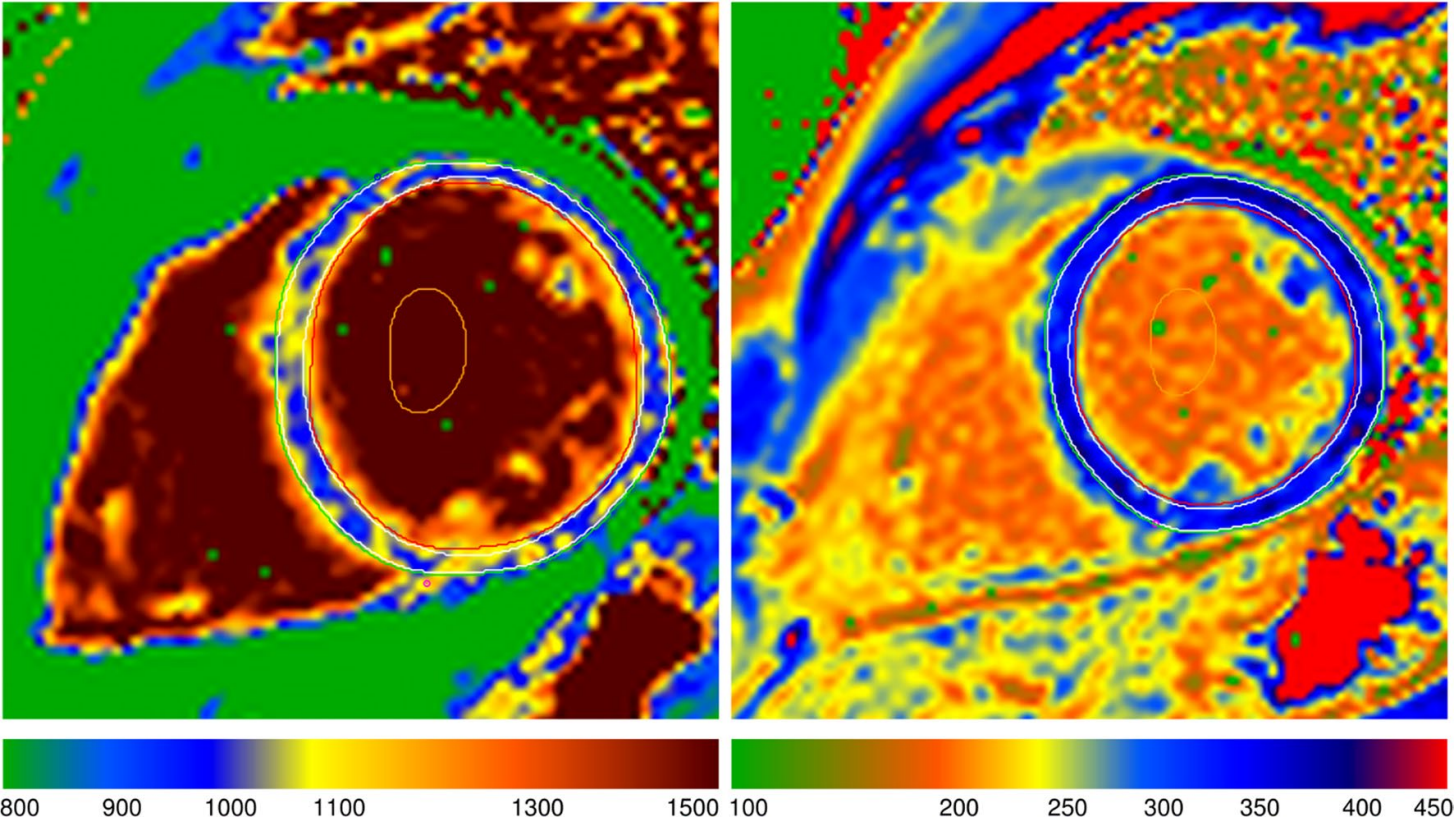


**Fig. 3** A representative picture of the native and post-gadolinium T_1_ map of DMD-FC. DMD-FC—Female carriers of Duchenne muscular dystrophy gene mutations

Figure 4 was incorrect, and it is shown below:
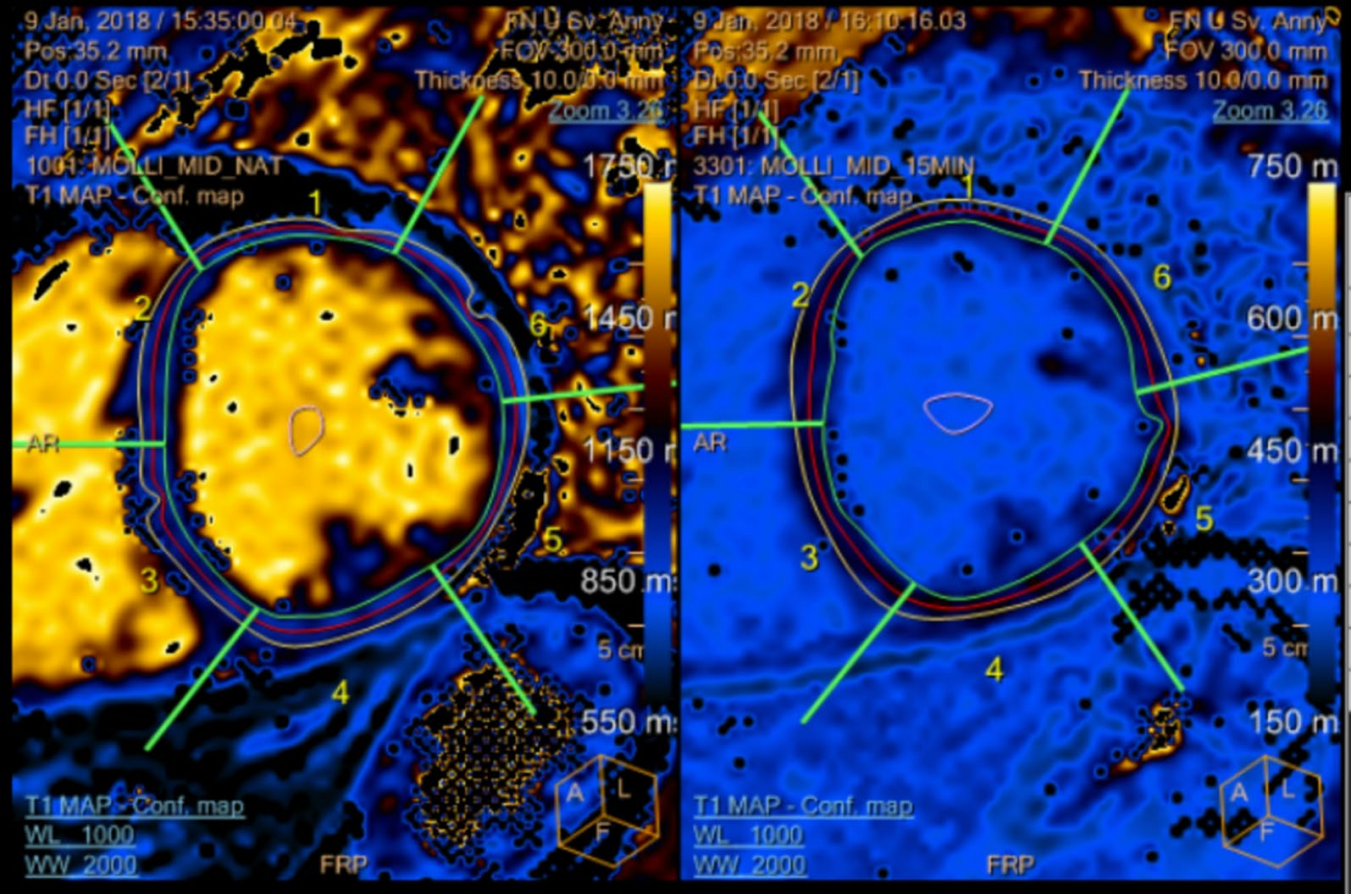


**Fig. 4** A representative picture of the native and post-gadolinium T_1_ map of a healthy volunteer

Figure 4 should be as follows:
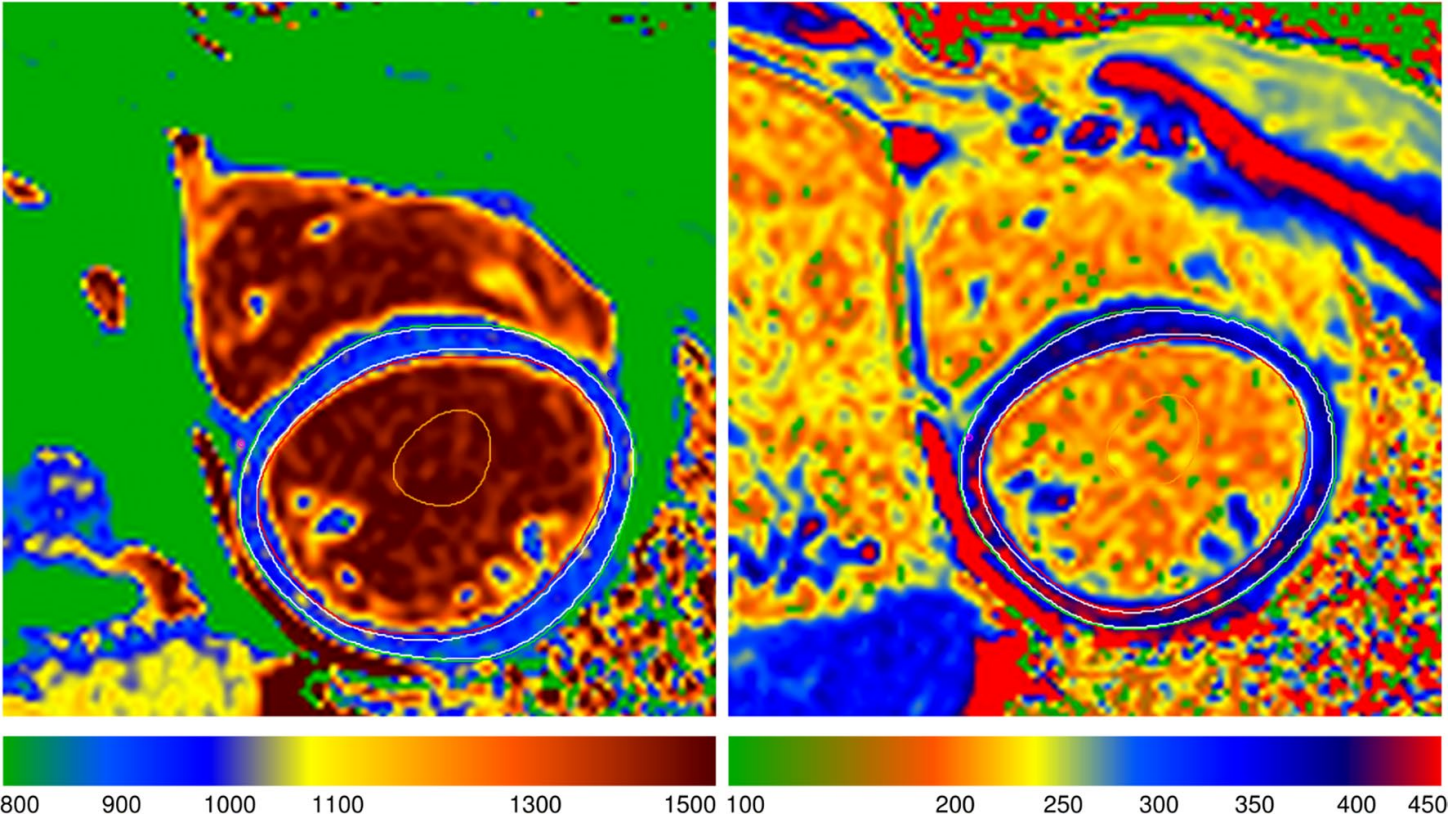


**Fig. 4** A representative picture of the native and post-gadolinium T_1_ map of a healthy volunteer

CMR acquisition section last paragraph had some incorrect spacing, it was as follows:

T_1_ mapping was performed as described previously [17] using a Modified Look-Locker Inversion recovery sequence (MOLLI) with a 5(3) 3 scheme to measure native T_1_ (pre-contrast) and a 4(1) 32 scheme for T_1_ post-gadolinium (15 min after contrast agent administration).

CMR acquisition section last paragraph text should be as follows:

T_1_ mapping was performed as described previously [17] using a Modified Look-Locker Inversion recovery sequence (MOLLI) with a 5(3)3 scheme to measure native T_1_ (pre-contrast) and a 4(1)3(1)2 scheme for T_1_ post-gadolinium (15 min after contrast agent administration).

The original article was updated.
