# Supplementary material for: Myocardial native T1 mapping and extracellular volume quantification in asymptomatic female carriers of Duchenne muscular dystrophy gene mutations
Source: Orphanet J Rare Dis. 2023 Sep 11;18:283. doi: 10.1186/s13023-023-02899-9 (PMC10496385; doi:10.1186/s13023-023-02899-9)
Supplement: Supplementary file 1 — Supplementary Material 1 [file 13023_2023_2899_MOESM1_ESM.docx]

**Answers to Reviewer`s Comments**

**Reviewer #1:**

*The authors appropriately responded to the reviewers' comments.*

Thank you very much for your positive evaluation of our revised manuscript.

**Reviewer #2:**

*The authors have improved the quality and readability of their manuscript.
They answered most points that I raised during the first review.*

Thank you very much for your positive assessment of our revised manuscript.

*Minor points remain:

1/ Few typos: e.g. "T1 relation" at several places*

We are sorry for this mistake. Of course „T_1_ relation“ has been changed to „T_1_ relaxation“.	“Also, there have been a few published reports about mildly elevated native T_1_ relaxation time or elevated ECV values in DMD/BMD-FC.”	“Moreover, we focused on the assessment of more than just the presence of LGE in the typical location or the described mildly elevated value of ECV or global native T_1_ relaxation time.”

*2/ Acronyms: DMD-FC should be used instead of FC at several places (except when authors mention BMD-FC) => this should be explicitly stated.*

Thanks for this point. DMD-FC or DMD/BMD-FC have been applied throughout the manuscript.

*3/ Acronym CG is used both for designing the 'control group' and 'healthy volunteer' subjects. In some sentences, it sounds very awkward: e.g. "The demographically similar study population included 38 DMD-FC with verified genetic mutation and 22 CG". 22 healthy volunteers were scanned, not 22 control groups…*

Of course, we transformed it the following form:	“38 DMD-FC with verified genetic mutation and 22 healthy volunteers were included.” “There were 38 DMD-FC (mean age 39.1 ± 8.8 years) and 22 healthy volunteers (mean age 39.9 ± 12.6 years) imagined by CMR.”	“The demographically similar study population included 38 DMD-FC with verified genetic mutation and 22 healthy volunteers.”	“The volunteers had no pathological findings on CMR, no anamnesis of cardiac disease, and no other pathological tests.”

*4/ Sequence description: 'repetition time' should be used instead of 'time to repetition'. Why TE/TR are given with the approximately equal sign?*

TE/TR values are equal due to the setting of our CMR device that measures the shortest value – the CMR device calculates the shortest value as is possible to obtain. It is the reason why the TE/TR values are usually very similar.

We also modified “time to repetition” to “repetition time”.

*5/ Paragraph "Wall motion abnormalities […] (SCMR) (21)." belongs to the section MR data analysis.*

The paragraph was written in the “MR data analysis” section.

*6/ MOLLI sequence description: 5s(3s)3s and 4s(1s)3s(1s)2s => is not the usual way to present the sequence scheme. Waiting time is in heartbeats and not in seconds. 5(3)3 and 4(1)3(1)2 are fine.*

We deleted the seconds from the scheme according to your suggestion:	“T_1_ mapping was performed as described previously using a Modified Look-Locker Inversion recovery sequence (MOLLI) with a 5(3)3 scheme to measure native T_1_ (pre-contrast) and a 4(1)3(1)2 scheme for T_1_ post-gadolinium (15 min after contrast agent administration).”

*7/ Define the acronyms in the formula for ECV calculation*

We explained the abbreviations in the formula for ECV quantification.

*8/ Figure 3-4 provide both native and post Gd T1 maps on a healthy control and DMD-FC. Please correct accordingly the text and figure legend*

Thank you very much for this valuable suggestion, the legends have been modified according your note.

*9/ Discussion : "There were no statistically significant differences in the mean global native T1 relaxation time or the mean global and segmental ECV values (…)". Global AND segmental native T1 relaxation time. => This also lacks in the conclusion.*

We completed and rephrased the discussion and conclusion following your point.
